# AI illuminates paths in oral cancer: transformative insights, diagnostic precision, and personalized strategies

**DOI:** 10.17179/excli2024-7253

**Published:** 2024-09-03

**Authors:** Devesh U. Kapoor, Pushpendra Kumar Saini, Narendra Sharma, Ankul Singh, Bhupendra G. Prajapati, Gehan M. Elossaily, Summya Rashid

**Affiliations:** 1 Dr. Dayaram Patel Pharmacy College, Bardoli-394601, Gujarat, India; 2 Department of Pharmaceutics, Sri Balaji College of Pharmacy, Jaipur, Rajasthan-302013, India; 3 Faculty of Pharmacy, Department of Pharmacology, Dr MGR Educational and Research Institute, Velapanchavadi, Chennai-77, Tamil Nadu, India; 4 Shree S. K. Patel College of Pharmaceutical Education and Research, Ganpat University, Kherva-384012, Gujarat, India; 5 Faculty of Pharmacy, Silpakorn University, Nakhon Pathom 73000, Thailand; 6 Department of Basic Medical Sciences, College of Medicine, AlMaarefa University, P.O. Box 71666, Riyadh, 11597, Saudi Arabia; 7 Department of Pharmacology & Toxicology, College of Pharmacy, Prince Sattam Bin Abdulaziz University, P.O. Box 173, Al-Kharj 11942, Saudi Arabia

**Keywords:** oral squamous cell carcinoma, machine learning, convolutional neural network, computed tomography, predictive modeling

## Abstract

Oral cancer retains one of the lowest survival rates worldwide, despite recent therapeutic advancements signifying a tenacious challenge in healthcare. Artificial intelligence exhibits noteworthy potential in escalating diagnostic and treatment procedures, offering promising advancements in healthcare. This review entails the traditional imaging techniques for the oral cancer treatment. The role of artificial intelligence in prognosis of oral cancer including predictive modeling, identification of prognostic factors and risk stratification also discussed significantly in this review. The review also encompasses the utilization of artificial intelligence such as automated image analysis, computer-aided detection and diagnosis integration of machine learning algorithms for oral cancer diagnosis and treatment. The customizing treatment approaches for oral cancer through artificial intelligence based personalized medicine is also part of this review.

See also the graphical abstract(Fig. 1).

## Introduction

Oral cancer ranks among the top 10 widespread cancers globally, often diagnosed at later stages. Regrettably, there are no exact biomarkers available for early detection, and treatment. The available options are costly, posing substantial challenges for patients. Oral squamous cell carcinoma (OSCC) is the standard term used to describe oral cancer, a malignancy that starts in the lip or oral cavity oral cancer (Rivera, 2015[86]). 

Oral cancer refers to the growth of cancerous cells in the mouth, including the lips, gums, tongue, cheeks, and roof of the mouth. It usually appears as stubborn sores or atypical growths in the mouth that don't heal on their own. This circumstance demands careful consideration because of its potential to profoundly affect oral health and overall well-being (Inchingolo et al., 2020[44]). 

The understanding of oral cancer involves various aspects like where it's located in the mouth, such as the lips, tongue, gums, or palate. The oral cancers generally appear as squamous cell carcinomas, although there are variations like adenocarcinomas and melanomas. The determination of cancer stage is important, as it helps distinguish the tumors based on their size, how far they've spread locally. The assessment of tumor grading is vital as it helps to understand the severity of cellular irregularities and the level of aggressiveness in cancer. The oral cancer classification is significantly affected by diverse factors such as, alcohol consumption, HPV infection, using tobacco, chewing betel quid. It is necessary to understand these classifications to make sure accurate diagnosis, evaluate prognosis, and prepare the customized treatment plans for those impacted by oral cancer (Warnakulasuriya et al., 2007[106]).

Tsu-Hui et al. anticipated that integrating tumor thickness into the TNM system could surge the prognostic accuracy. Tumors underwent reclassification, with those ≤5 mm thick or ≤4 cm in diameter now categorized as T1, while those >5 mm thick or still ≤4 cm in diameter were selected as T2. In the latest TNM classification system, survival rates show no prominent contrast between the T1 and T2 categories for disease specific survival (p = 0.16) or overall survival (p = 0.69), representing alike outcomes for patients in both the groups (Low et al., 2015[62]).

The epidemiology of oral cancer consists of analyzing its occurrence, prevalence, distribution, and contributing factors within populations. Oral cancer ranks among the top 15 most prevalent cancers globally, with fluctuating rates observed across dissimilar geographical areas and demographic groups. While affecting both the genders, men mainly experience a more incidence compared to women (García-Martín et al., 2019[32]). The risk factors for oral cancer encompass tobacco use in different forms (smoking and smokeless products), extreme alcohol consumption, chewing of betel quid, and infection with human papillomavirus (HPV), particularly HPV-16. The disproportions in oral cancer occurrence among the diverse regions can be attributed to cultural norms, socioeconomic conditions and healthcare accessibility (Conway et al., 2018[19]). The imperative strategies for decreasing the oral cancer burden include early finding through routine screenings and growing awareness about risk factors (Fernández et al., 2015[30]).

The current diagnostic methods for oral cancer comprise an amalgamation of clinical examination, imaging approaches (Pérez et al., 2015[79]) and tissue biopsy (Gopinath et al., 2021[34]). The healthcare specialists examine the oral cavity for any oddities such as sores, red/white patches, or lumps at the time of clinical examination. The cutting-edge imaging techniques like computed tomography (CT) scans, positron emission tomography (PET) scans (Keshavarzi et al., 2017[51]) and magnetic resonance imaging (MRI) may be employed to characterize the extent of tumor growth, invasion into adjacent tissues, and potential spread to nearby lymph nodes. The tissue biopsy remains the main procedure for confirming the incidence of cancerous cells. In this technique, extracting a small tissue sample from the infected lesion and inspecting it under a microscope for the presence of malignant cells. For the early oral cancer detection and diagnosis, emerging technologies include optical imaging (Malik et al., 2016[64]) and molecular biomarkers (Cervino et al., 2019[14]), are also being explored. The quick development in diagnostic techniques aims to intensify the accuracy, efficiency, and patient outcomes in oral cancer treatment.

### Role of imaging in oral cancer

#### Traditional imaging techniques

The traditional imaging techniques for oral cancer detection comprise non-invasive techniques such as X-rays, CT (Mukherji et al., 2001[73]), MRI (Neto et al., 2018[76]), and ultrasound. These techniques offer thorough images of the oral cavity and adjacent structures, provide support in the visualization of tumors, their size, extent of invasion, and involvement with the lymph nodes. The X-rays are a rapid and cheap option to recognize abnormalities in the bones of the jaw and teeth. The CT scans also employ the X-rays to generate cross-sectional pictures, offering the valuable information about the precise location and spread of tumors. The MRI technique uses magnetic fields and radio waves to produce high-resolution images, mainly useful for characterizing soft tissue structures like the tongue and floor of the mouth (Reda et al., 2021[84]). The ultrasound imaging uses the sound waves to generate real-time images, supporting in the significant evaluation of superficial lesions and lymph nodes (Guo et al., 2018[36]). These conventional imaging methods play a critical role in the diagnosis and treatment of oral cancer. These techniques offer quick detection and treatment planning while diminishing patient risk and discomfort (Chakraborty et al., 2019[15]).

#### Limitations of conventional imaging

There are some limitations with the conventional imaging techniques in the detection of oral cancer. The X-rays, although very useful for assessing the bone abnormalities, may not sufficiently be able to visualize soft tissue lesions (Murray, 2015[74]). The CT scans offer the thorough pictures of the oral cavity but comprise ionizing radiation, which can pose dangers, mainly with recurrent exposure (De Felice et al., 2019[21]). The MRI offers exceptional soft tissue contrast but may be less available and more time consuming as compared to other imaging techniques (Ahlawat et al., 2019[2]). MRI may not consistently distinguish between benign and malignant lesions. The ultrasound imaging is limited by its inability to enter deeply into tissues, constraining its utility in evaluating deeper structures and metastatic lymph nodes. All these techniques generally offer anatomical information and may not constantly and precisely distinguish between benign and malignant lesions. So, while conventional imaging plays an important role in diagnosis of oral cancer, its limitations accentuate the need for complementary methods and developing technologies to surge early detection and improve patient outcomes (Singhal et al., 2023[94]).

#### Introduction to artificial intelligence in imaging

AI has appeared as a transformative tool in cancer imaging, transforming the way tumors are detected, diagnosed, and treated. With the help of machine learning algorithms, AI can analyze huge amounts of medical imaging data with incomparable speed and exactness, supporting radiologists in detecting subtle abnormalities indicative of cancer (Mandal et al., 2018[65]). Along with the deep learning techniques, AI models can recognize the patterns and features in images that may not be readily apparent to the human eye, leading to earlier and more accurate cancer diagnoses. The algorithms of AI can also predict the tumor growth, evaluate treatment response, and even support in personalized treatment planning by examining imaging data in combination with other clinical information (Liefaard et al., 2021[60]). The integration of AI into cancer imaging not only surges diagnostic accuracy but also enhances the patient outcomes by enabling timely interventions and custom-made therapeutic approaches. As AI continues to progress and mature, its role in cancer imaging is poised to expand further, giving new insights and innovations that will continue to transform the field of oncology (Iqbal et al., 2021[45]).

## Applications of Artificial Intelligence in Oral Cancer Diagnosis

**Figure 2:**
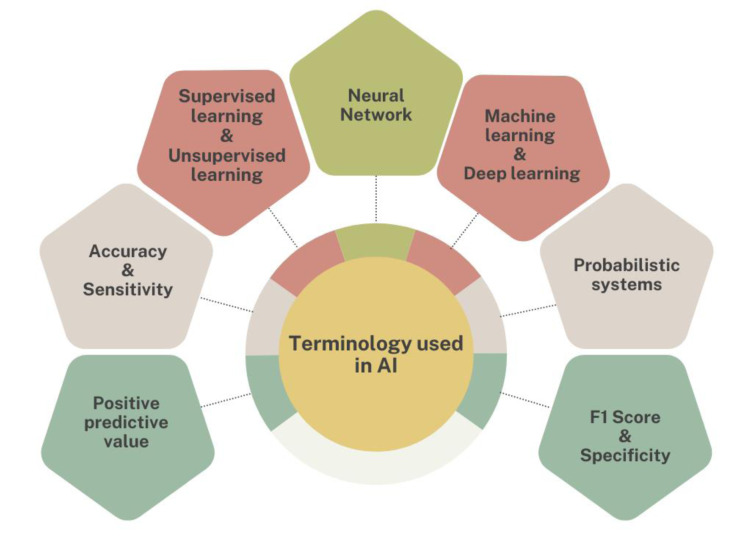
Various terms used in AI

There has been an increment in the occurrence of oral cancer globally, leading to escalated morbidity and mortality, mainly when detected at advanced stages. The utilization of technology could aid in initial detection and diagnosis, thereby simplifying better patient care and management for clinicians. The emergence of AI holds promises for enhancing oral cancer screening. AI can accurately examine vast datasets from diverse imaging techniques, offering valuable support in the oncology domain (Hegde et al., 2022[38]). The different terminologies employed for AI are depicted in Figure 2(Fig. 2).

### Automated image analysis

Automated image analysis holds substantial potential for the oral cancer diagnostics and treatment monitoring. This technology is utilizing sophisticated algorithms for exact identification of cancerous cells and tissue characteristics, simplifying initial detection and tailored therapeutic interventions (Webster and Dunstan, 2014[108]). Its integration into clinical practice gives the potential to surge diagnostic exactness, streamline workflows, and finally enhance the patient outcomes in the fight against oral cancer.

Multimodal optical imaging amalgamated with autofluorescence imaging and high-resolution microendoscopy for diagnosing oral cavity conditions. It supports to identify the pinpoint high-risk areas for potential neoplasia, suggesting a non-invasive and accurate diagnostic technique. 

Quang et al. conducted an examination of multimodal optical imaging joined with automated image analysis for the real-time recognition of oral neoplasia in living individuals. The researchers used 206 different locations and 100 patient samples were scanned to capture multimodal optical images. The 92 sites from the initial 30 patients served as the foundation for preparing automated image analysis methods to spot neoplasia. To assess diagnostic efficacy, 114 sites from the remaining 70 patients were used as a test set. The analysis revealed that multimodal optical imaging, coupled with automated methods, accurately distinguished nonneoplastic and neoplastic sites, achieving a success rate of 96 % and 95 %, respectively, in the training set. In the test set, multimodal optical imaging accurately identified all nonneoplastic sites and correctly classified 86 % of neoplastic sites from the 56 biopsied locations. When analyzing 58 sites corresponding to surgical specimens, it still achieved a perfect classification rate for nonneoplastic sites but identified neoplastic sites with a slightly lower accuracy of 62 %. The researchers noticed a significant correlation between elevated p63 expression, and sites graded as mild dysplasia, particularly those identified as neoplastic through optical assessment, with a prevalence of 60 %. However, among sites graded as mild dysplasia but categorized as nonneoplastic via optical assessment, only 27 % showed high p63 expression. The study suggests that combining various optical imaging techniques could surge the detection of oral neoplasia, aiding in more accurate diagnosis and guiding biopsy site selection (Quang et al., 2017[81]).

Yumii et al. explored the potential of employing radiomics, an AI driven image analysis approach, to exactly determine the stage of laryngopharyngeal cancer. Researchers incorporated esophagogastroduodenoscopic pictures of 95 lesions, confirmed as squamous cell carcinoma (SCC) through pathological examination, which underwent transoral surgery at their medical facility. Radiomics analysis was conducted on 95 upper gastrointestinal endoscopic NBI images of lesions, revealing 54 cases of SCC *in situ* and 41 cases of SCC among the 95 lesions examined. In the Radiomics research, the average cross-validation yielded a score of 0.845, while the mean AUC from the ROC curve stood at 0.869. These findings closely mirrored the diagnostic accuracy of a proficient endoscopist. Radiomics investigation exhibits promise in clinically applying the assessment of laryngopharyngeal cancer depth, suggesting significant diagnostic advancements (Yumii et al., 2024[115]).

Ariji and his colleagues assessed the efficacy of deep learning image classification for identification of lymph node metastasis. This assessment employed CT scans encompassing 128 confirmed positive cervical lymph nodes and 315 confirmed negative lymph nodes from 45 patients of OSCC. A deep learning system's ability to categorize lymph node metastasis on CT images was evaluated against interpretations from two radiologists. The statistical analysis which includes the Mann-Whitney U test and χ2 analysis, were employed to compare their diagnostic performance, guaranteeing noteworthy evaluation. The deep learning image classifier attained an accuracy of 79.5 %, with sensitivity at 76.8 % and specificity at 82.1 %. The positive predictive value stood at 80.2 %, while the negative predictive value reached 78.3 %. Its performance, indicated by an area under the receiver operating characteristic curve of 0.81, exhibited robustness in distinguishing target classes. The outcome was alike to those identified by the radiologists, exhibiting no substantial differences. The diagnostic outcomes of AI's meticulously mirrored those of radiologists, demonstrating its potential as an important tool for supporting in diagnoses (Yumii et al., 2024[115]).

### Computer-aided detection and diagnosis

In 2019, a groundbreaking investigation introduced a sophisticated computer-aided algorithm intended to support in oral cancer detection. This novel system examines hyperspectral images of patients' oral tissues, contributing automated and accurate diagnostic support. The researchers offered a new architecture for a partitioned convolutional neural network (CNN) intended mainly for specific medical image classification. This advanced approach employes two partitioned layers to pinpoint areas of interest within multidimensional hyperspectral images, increasing precision in analysis and diagnosis. The assessment of classification accuracy calculated the efficacy of the segmented deep CNN. The results revealed a 92.3 % accuracy rate, with 0.95 % sensitivity and 0.92 % specificity, using 100 image data for training in classifying benign and malignant tumors. In addition, classification of normal tissue versus cancerous tumors attained a 95.6 % accuracy with 500 training patterns. Researchers have determined that an advanced CNN learning algorithm, based on regression, enhances the accuracy of diagnosing complex cases of oral cancer, improving diagnostic quality considerably (Agarwal et al., 2023[1]).

### Integration of Machine Learning algorithms

The utilization of algorithms of ML in oral oncology aids in addressing diagnostic challenges, escalating accuracy, and recuperating patient outcomes. The systems employing ML enable the generation of predictions through accumulated experience from training, with algorithms establishing connections among the provided data (Tobias et al., 2022[98]).

Warin and his team of researchers explored an assessment of CNN models to categorize and recognize oral potentially malignant disorders (OPMDs) from the images of the mouth. In this investigation, 600 oral photographs were collected retrospectively, separated into two sets consisting of 300 images illustrating OPMDs and another 300 showing normal oral mucosa. The classification models were prepared employing DenseNet-121 and ResNet-50, whereas the detection models used Faster R-CNN and YOLOv4. DenseNet-121 and ResNet-50 exhibited significant efficacy in identifying OPMDs, attaining an impressive area under the receiver operating characteristic curve of 95 %. The quicker R-CNN model showed outstanding detection performance, attaining an AUC of 76.29 %. The CNN-based classification model, sensitivity reached to 100 % while specificity stood at 93 %. The corresponding figures stood at 92.58 % and 93.27 % respectively, among oral and maxillofacial surgeons (Warin et al., 2022[105]).

Sun and colleagues endeavored to authenticate a novel approach for forecasting the proliferation status of tongue squamous cell carcinoma (TSCC) via contrast enhanced CT (CECT) using CNN. The CECT scans of the lesion region from 179 TSCC patients underwent analysis utilizing a CNN. The patients were segregated into high and low proliferation status groups based on their Ki-67 index, with a cutoff set at the median value of 20 %. After training, the model automatically classified the test set, revealing an accuracy of 66.41 % and an AUC of 0.7234. These results indicate that a significant portion of samples were correctly classified, affirming the stability of the model. The researchers suggest the potential for non-invasive prediction of TSCC proliferation status using AI on contrast-enhanced CT scans prior to surgery (Sun et al., 2022[96]).

Sharma and colleagues investigated a deep learning algorithm utilizing CNN to distinguish between oral precancerous/cancerous lesions and normal mucosa. Existing CNNs are utilized to identify precancerous and cancerous lesions in the oral cavity, differentiating them from healthy mucosal tissue. This is achieved through the analysis of a dataset comprising photographic images that have been clinically annotated. Transfer learning using various pre-trained CNN architectures was utilized for image classification tasks, resulting in accuracy rates of 77 % using VGG19, 73 % for VGG16, and 73 % with MobileNet. InceptionV3 achieved an accuracy of 69 %, while ResNet50 demonstrated a lower accuracy of 35 %. Among these models, VGG19 demonstrated superior performance in the current investigation compared to others (Sharma et al., 2022[92]).

Welikala and colleagues evaluated two advanced computer vision methodologies for detecting and categorizing oral lesions, aiming to facilitate early identification of oral cancer. Their research focused on leveraging deep learning techniques to enhance automated systems for swift and accurate diagnosis, potentially improving patient outcomes through timely intervention. The investigators utilized ResNet-101 for image classification and Faster R-CNN for object detection. The image classification model achieved an F1 score of 88.12 % for identifying images with lesions and 79.58 % for identifying images needing referral, showcasing its effectiveness in medical image analysis. The object detection system successfully identified lesions needing referral with an F1 score of 42.28 % (Welikala et al., 2020[109]). 

Esce et al. utilized CNN to forecast nodal metastases in oral TSCC. The researchers employed AI/HALO CNN along with image software for training and testing purposes. The AI/HALO method analyzed the cases of the test set without bias, assigning a likelihood score to each region (patch) for the most probable assessment of nodal status. The algorithm classifies regions as either red or green based on lymph node status, indicating positivity or negativity respectively. This results in a mosaic pattern of red and green regions for each case, as determined by the predictive model. The outcomes for each test scenario were measured as the percentage of the identified positive area, termed as the area distribution percentage (AD %). Primary analysis involved utilizing receiver operator characteristic (ROC) curves and the Youden J statistic. The study encompassed eighty-nine instances of squamous cell carcinoma affecting the oral tongue. The most effective algorithm, under rigorous supervision, demonstrated a 66 % sensitivity and 87 % specificity in detecting nodal metastases. The ROC curve's area under the curve for this algorithm was measured at 0.738. A CNN can generate an algorithm capable of forecasting nodal metastases in individuals with oral TSCC solely through analysis of the visual histopathology of the primary tumor (Esce et al., 2024[27]).

Monreal and colleagues assessed multiple machine learning algorithms to forecast the survival time of ovarian cancer patients, identifying the model demonstrating superior performance for prospective integration into clinical practice. Twenty distinct machine learning algorithms were utilized to analyze a dataset split into 70 % for training and 30 % for testing purposes. Following this, the Recall, F-1 score, and Importance Score (IS) were computed. A cohort of 703 individuals diagnosed with oral leukoplakia participated in the study, comprising 45 % females and 84 % identifying as White or Caucasian ethnicity. The average age upon diagnosis of oral leukoplakia stood at 60 years, with 57 % reporting no history of smoking, and 64 % indicating they were in a partnership. Within the initial year following the oral leukoplakia diagnosis, 58 patients progressed to develop oral cancer. Histological identification of oral leukoplakia was evident in 15 % of patients and indicated dysplasia preceding the diagnosis of oral cancer. Tuned random forest and tuned decision Tree emerged as the most effective machine learning algorithms, excelling in specificity and accuracy. Both models exhibited around 62 % accuracy in forecasting oral cancer in patients with oral leukoplakia, with a recall (specificity) surpassing 82 %. Key influential variables included "age under 40 years," "prior smoking habit," "White or Caucasian ethnicity," never smoking, "presence of leukoplakias elsewhere," "being partnered," and "history of oral dysplasia” (Monreal et al., 2023[71]).

Another study carried out by Het and colleagues, utilized continuous DNA aneuploidy cytology, assisted by artificial intelligence, to monitor dysplastic oral leukoplakia undergoing photodynamic therapy. During the oral examination, a reappearance of a dissimilar white patch, approximately 8 mm × 5 mm in dimensions was observed on the underside of the tongue's left side. DNA aneuploidy cytology by image cytometry (DNA-ICM) with AI assistance revealed the presence of a single aneuploid cell. The patient received the second 5-aminolevulinic acid-PDT session weekly, totalling three treatments. Temporary erosion and hyperemia occurred at the treatment site, resolving completely one month post the second session. Currently, there were no aneuploid cells detected, and there was no reappearance observed during the 5-month follow-up following the second 5-aminolevulinic acid-PDT session. The DNA-ICM analysis with AI assistance revealed the absence of DNA aneuploid cells (He et al., 2023[37]).

Sure, here's a humanized and plagiarism-free paraphrase of the content:

Shamin and his team utilized TinyML, AI based ML to detect oral tongue lesions early, enhancing healthcare accessibility. The planned visual system uses a carefully labeled photo collection of nine different kinds of surface-level tongue lesions to enhance a MobileNetV2 neural network through transfer learning. The researchers employed TensorFlow Lite for Microcontrollers to convert a high-precision model into a low-precision one, suitable for deployment on a resource-limited OpenMV Cam H7 Plus. The specialized int8 model precisely identified nine tongue lesions with a 99.24 % success rate during testing. Compared to the float32 model, it achieved similar inference speeds (1.1 ms) on the edge device while reducing on-device RAM and flash memory usage by over 60 % (Shamim, 2022[91]).

Junior pathologists experienced a 6.26-minute reduction in oral cancer delineation time when aided by the deep-learning model, as per findings by Yang et al. The temporal aspect carries significant implications for cancer centers experiencing heavy workloads. Developing a ML model to address the research gap in oral OSCC by considering the time factor crucial during intra-operative frozen section analysis of surgical margins is imperative (Yang et al., 2022[113]). 

James and colleagues utilized advanced computational techniques, like artificial neural networks and support vector machines, to analyze image characteristics of Optical coherence tomography (OCT) scans from healthy oral tissue and both benign and malignant lesions. Studies revealed that using OCT-based diagnosis alongside AI for identifying malignant and dysplastic oral lesions showed similar effectiveness (93-96 % sensitivity and 74-49 % specificity) as traditional biopsy methods, indicating promising potential for non-invasive diagnostic approaches (James et al., 2021[46]). 

Alhazmi and colleagues explored ANN model to forecast an individual's likelihood of developing oral cancer. Their approach involved analyzing various factors like risk elements, medical history, and clinical characteristics to enhance predictive accuracy and aid in early detection. The researchers found that the neural network's ability to predict oral cancer, assessed through 10-fold cross-validation, yielded an average sensitivity of 86.69 % and specificity of 59.89 %. The ANN achieved a prediction accuracy of 79.58 % in diagnosing oral cancer (Alhazmi et al., 2021[5]).

The summary of different AI models employed for detection and treatment of oral cancer is illustrated in Table 1(Tab. 1) (References in Table 1: Cai et al., 2023[12]; Das et al., 2023[20]; Huang et al., 2024[41]; James et al., 2021[46]; Jubair et al., 2022[49]; Krisha and Peram, 2023[55]; Marzouk et al., 2022[67]; Rahman et al., 2020[83]; Sharma et al., 2022[92]; Warin et al., 2022[105]).

## AI in Oral Cancer Prognosis and Prediction

AI, by analyzing diverse medical images and relevant factors such as age, gender, and biomarkers, outperforms conventional methods in diagnosing and predicting ovarian cancer, showing higher precision and accuracy than current clinical strategies and statistical analyses. AI is better at detecting oral cancer than traditional methods (Verghese et al., 2023[102]). Analyzing past patient data can enhance AI diagnosis. Early detection is crucial for effective treatment, given the challenging investigation due to complex causes and high recurrence rates where AI helps classify patients into high and low-risk groups, aiding clinicians in planning treatment better than conventional methods. This ensures patients receive sound advice, and clinicians make informed decisions (Khanagar et al., 2021[53]; Tiwari et al., 2023[97]). Oral potentially malignant disorders frequently serve as the initial indicators of oral cancer, though predicting the progression of these cases into malignancies poses a challenge. Traditional diagnostic methods, such as visual oral examination and histological examination, remain the standard initial procedures for identifying oral lesions. Nevertheless, these approaches have inherent limitations that may result in delayed diagnoses of oral cancer or overlooked identification of disorders in individuals at high risk. Contemporary innovative methods, including liquid biopsy, next-generation sequencing, microarray, nanotechnology, lab-on-a-chip or microfluidics, and artificial intelligence, are presently employed for the clinical diagnosis and treatment of this malignancy (Dholariya et al., 2023[23]). Recurrence of oral cancer after surgery significantly affects the patient's prognosis. A machine learning model has been developed to predict the probability of postoperative recurrence in patients with oral cancer using artificial intelligence. The multidimensional Perceptron model exhibits high sensitivity to predict oral cancer recurrence after surgery, with models trained on variables proving superior using feature variable inputs (Cai et al., 2023[12]). Therefore, prognostic prediction of cyclin D1 expression patterns in HPV-negative oral oropharyngeal cancers is examined using a deep learning approach. Diagnosis of oral oropharyngeal squamous cell carcinoma incorporating image recognition patterns for cyclin D1 expression patterns of the incidence of infected patients has some effective prognostic impact on negative overall survival for human papillomavirus (Yang et al., 2023[112]).

### Predictive modeling

In the realm of oral cancer, a deep learning predictive technique is employed using automated assessment of masseter muscle volume (Sakamoto et al., 2024[89]). Building a learning model for masseter muscle volume is vital, as indicated by the substantial relationship identified between the life expectancy for individuals with oral cancer and manually extracted masseter muscle volume on CT scans. Since life expectancy is substantially forecasted by the automatically recovered masseter muscle volume using the prevailing model, the sarcopenia evaluation approach can be beneficial in assessing life expectancy for individuals with oral cancer. A distinctive deep learning-based approach has been established to forecast longevity in instances of oral cancer through investigating the immune cell concentration in tumors. The scientists implemented a clustering technique utilizing CIBERSORT profiles from openly accessible data in The Cancer Genome Atlas and survival patterns were sought in accordance with clustering scores. Identifying similar genes, transcribed differentially, emphasizes the essential role of tumor-infiltrating lymphocytes in the tumor microenvironment that offers a feasible approach towards applying deep learning to cancer prognosis (Kim et al., 2021[54]). AI models are often employed for diagnosing oral cancer and used to categorize the complexity of the illness, anticipate patient prognosis, and differentiate between normally functioning and cancerous sections. It can enhance precision and accuracy, aiding pathologists with providing a more precise assessment and fewer mistakes (Khanagar et al., 2023[52]). The risk prediction models for OSCC that use machine learning and salivary autoantibody biomarkers are verified using a variety of algorithms, including stacking models, random forests, logistic regression, support vector machines with the radial basis function kernel, Extreme Gradient Boosting, and random forests thereby designed for clinical usage with the goal of aiding in the diagnosis of OSCC while possibly lowering disease morbidity and death rates (Tseng et al., 2022[100]). Prior to surgery, an innovative method for predicting the rate of growth of tongue squamous cell carcinoma can be assessed by combining AI with contrast-enhanced CT (CECT) by utilizing Convolutional neural networks to interpret CECT images from squamous cell carcinoma patients. The patient's classification is kept as high or low proliferation status based on their Ki-67 index. The test results provide higher accuracy rate for the AI model that implies a possible non-invasive technique for anticipating the stage of cell cancer proliferation prior to surgery (Sun et al., 2022[96]). 

**Figure 3:**
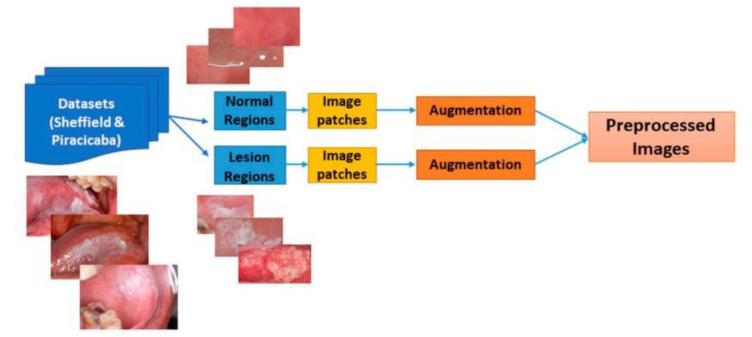
Image preprocessing block diagram. Two datasets contain annotated images cropped from bounding boxes delineating suspicious and normal regions. After those two images are converted into 128x128 image patches. Finally, image of patches was enhanced (Figure taken from Camalan et al., 2021, CC BY license).

Camalan and colleagues devised a clinical predictor of oral dysplasia utilizing CNN. The researchers employed transfer learning with a CNN technique, utilizing the Inception-ResNet-v2 pre-trained model and analogized outcomes with VGG-16 and ResNet-101 models. An approach was employed to differentiate images as either "suspicious" or "normal" utilizing transfer learning with Inception-ResNet-V2. Before determining whether an image is normal or suspicious, the system conducts a preprocessing stage to ready the images for classification depicted in Figure 3(Fig. 3) (Reference in Figure 3: Camalan et al., 2021[13]). Moreover, automated heat maps were created to pinpoint areas within the images likely prompting the decision-making process. The system underwent rigorous testing employing both 10-fold cross-validation and leave-one-patient-out validation methods. The outcome revealed the accuracies of 74.7 % (±20 %) and 91.4 % (±13 %), F1-scores of 98.2 % and 88.3 %, and precision values of 94.5 % and 98.7 % (Camalan et al., 2021[13]).

**Figure 4:**
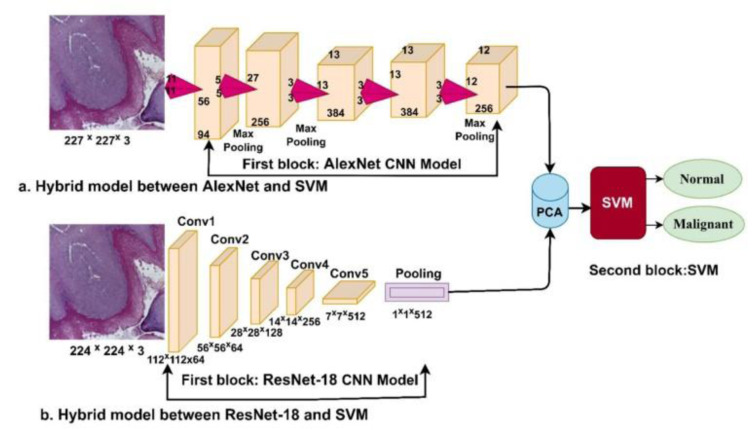
A hybrid technique, merging CNN and SVM models, is used for histopathological image diagnosis of oral cancer (Figure taken from Fati et al., 2022, CC BY license).

**Figure 5:**
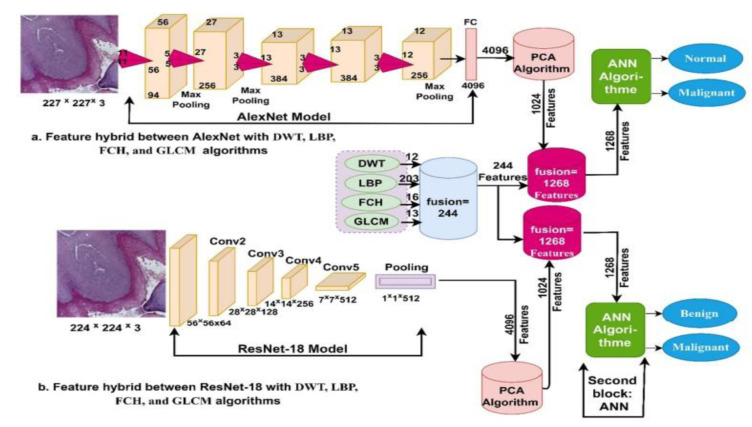
A novel method utilizing a hybrid features technique integrating CNN model, DWT, LBP, FCH, and GLCM modules for histopathological image diagnosis of oral cancer (Figure taken from Fati et al., 2022, CC BY license).

Fati and colleagues investigated the early detection of OSCC via histopathological image analysis employing advanced deep and hybrid learning methodologies. The initial method encompasses CNN models (AlexNet and ResNet-18) with the SVM algorithm, establishing a hybrid approach depicted in Figure 4(Fig. 4) (Reference in Figure 4: Fati et al., 2022[29]). This approach established excellent outcomes in diagnosing the OSCC dataset. The alternative method consists of hybrid features from CNN models (AlexNet and ResNet-18) with color, texture, and shape attributes extracted via fuzzy color histogram (FCH), discrete wavelet transforms (DWT), local binary pattern (LBP), and gray-level co-occurrence matrix (GLCM) algorithms for improved feature representation. (Figure 5(Fig. 5); Reference in Figure 5: Fati et al., 2022[29]). The combination of ResNet-18 and SVM outperforms AlexNet with SVM, with ResNet-18 + SVM attaining significant accuracy (98.5 % vs. 96.3 %), specificity (98.54 % vs. 96.53 %), sensitivity (98.34 % vs. 96.12 %), precision (98.67 % vs. 96.78 %), and AUC (97.89 % vs. 98.11 %). An ANN employing hybrid features from AlexNet, DWT, LBP, FCH, and GLCM achieved outstanding performance, with a precision of 99.4 %, specificity of 99.69 %, sensitivity of 99.6 %, precision of 99.45 %, and AUC of 99.69 %. Likewise, utilizing hybrid features from ResNet-18, DWT, LBP, FCH, and GLCM, the ANN attained a precision of 99.4 %, specificity of 99.39 %, sensitivity of 99.56 %, precision of 99.42 %, and AUC of 99.45 % (Fati et al., 2022[29]).

### Identification of prognostic factors

A highly supervised CNN is employed to show nodal metastases in oral tongue squamous cell carcinoma with better sensitivity and specificity but still requiring additional approaches to be employed in therapeutic settings (Esce et al., 2024[27]). A novel deep learning model was created to enhance the diagnosis of OSCC from histopathology images. It was demonstrated that using this approach could significantly improve photo categorization speed and accuracy. In comparison, junior pathologists required 6.26 minutes less time to identify cancer cells when working with the model suggesting the improvement in deep learning algorithms using the histopathological image based OSCC diagnosis process (Yang et al., 2022[113]). Sarcopenia is considered to be one potential factor influencing the prognosis of cancer patients. An approach aims in establishing the relationship between cancer patients' prognosis and sarcopenia as determined by masseter muscle volume on CT scans. A deep learning algorithm that automatically extracts the masseter muscle volume has been utilized in order to evaluate the life expectancy of individuals with oral cancer further warranting the comparison of the anticipated lifespan of the patients (Sakamoto et al., 2024[89]). Immunohistochemistry is widely used by scientists to analyze the expression of β-catenin and its correlation with PD-L1, the number of lymphocytes and macrophages, and other variables in order to forecast the progression of OSCC to acknowledge its association with the immune system. Individuals with membranous β-catenin in the tumor and high levels of stromal CD8+ T-cell infiltration are predicted to have a positive prognosis for OSCC patients (Lequerica-Fernández et al., 2023[58]). Thus, AI based classification system by employing CNN is utilized for multiclass grading of OSCC and segmenting of epithelial and stromal tissue. Interesting research on salivary exosome offers an in-depth and non-invasive technique that can be employed to recognize oral cancer and predict how it will progress. Research indicates that the characteristics of these exosomes can be used as reliable markers to differentiate between tobacco users and non-smokers as well as oral cancer patients. This indicates how exosomes may be used to identify oral cancer in high-risk individuals early on (Bano et al., 2023[9]). IL17RB is a major player in the development of lung cancer. It was found that clinicopathological characteristics were related to its expression in patients with OSCC. High IL17RB expression in OSCC tissues, compared to normal oral mucosa tissues, was positively connected with factors like tumor size, lymph node metastasis, advanced cancer stage, and poor prognosis. This suggests IL17RB's potential as an indicator of progression-free survival and response to radiotherapy and chemotherapy in OSCC patients (Yuan et al., 2024[114]). Differences in how cancers respond to treatment and how long patients survive are linked to variations in immune contextures. When there's a lot of anti-tumor immune cells in both the tumors and nearby areas, it often leads to a positive prognosis. On the other hand, if there's minimal infiltration in tumors despite high infiltration in surrounding areas, it tends to result in a poorer prognosis. Using targeted CD73 immune-checkpoint inhibition could potentially enhance clinical outcomes in these cases (Chatterjee et al., 2023[17]). Analyzing data from a database of early-stage OSCC patients without affected lymph nodes revealed that poor differentiation significantly affects survival. Poor differentiation alone can impact survival in early oral cancer, particularly in patients with tongue cancer and potential associated perineural invasion (Mathur et al., 2023[68]). 

### Risk stratification

The K-nearest-neighbor algorithm in machine learning is recently adapted to predict oral cancer survival time and classify stages. It employs regression for predicting survival time and classification for categorizing patients into predefined stages, using hold-out and k-fold cross-validation methods. Observations indicate that older patients with more mutations face higher short-term survival risk, while seniors with significant mutations have an increased chance of reaching the last cancer stage (Siddalingappa and Kanagaraj, 2022[93]). Autoantibodies linked with tumors can act as signs to identify various cancers. Early risk prediction of OSCC is aided by the use of machine learning algorithms using salivary biomarkers. For high-risk OSCC cases, this could be extremely helpful in directing clinical treatment and prognosis (Wang et al., 2021[104]). The process of grouping individuals based on their likelihood of developing oral cancer or experiencing adverse effects from the disease is called risk stratification. Applying AI to risk stratification, which analyzes complex datasets using state-of-the-art technology, is expected to yield ground-breaking findings containing genetic data, imaging results, patient histories, and other relevant data (Roberts et al., 2021[87], Beaulieu-Jones et al., 2021[10]). AI could possibly be equipped recognizing patterns and relationships in these datasets that are not readily apparent when using traditional methods (Wong et al., 2021[110]). Additionally, the accuracy of diagnoses can be strengthened by implementing AI in risk stratification (Liao et al., 2022[59]). By processing massive quantities of data quickly and reliably, artificial intelligence (AI) systems can aid in the early detection and diagnosis of oral cancer, which is important for putting timely therapies into place and improving patient outcomes (Hunter et al., 2022[42]). 

## AI in Oral Cancer Personalized Medicine

### Tailoring treatment strategies with AI

Research is employed to forecast the outcome of oropharyngeal cancer treatment after surgery using MRI scans and machine learning through the analysis of preoperative MRI scans to extract radiomics features. It can be attributed by building a predictive model for illness recurrence and mortality that combine clinical parameters and MRI radiomics features, demonstrating stronger performance. However, a multicenter investigation can be implemented in the future to confirm and evaluate the model's therapeutic usefulness (Min Park et al., 2021[70]). Patient phenotypes with distinct healthcare requirements or uncommon therapeutic responses are identified through precision medicine techniques. AI, by utilizing sophisticated computational and inference skills, not only produces insightful outputs but also empowers the system to learn and reason on its own. This augmented intelligence empowers clinicians, enhancing their decision-making processes (Johnson et al., 2021[48]). The promising accuracy demonstrated by artificial intelligence in diagnosing and predicting oral cancer suggests a potential breakthrough in leveraging advanced technology for improved healthcare outcomes (Baniulyte and Ali, 2022[8]). Empirical therapies for oral cancer, encompassing surgery, radiotherapy, and chemotherapy, exhibit varied efficacy and entail side effects, yielding a bleak prognosis. Urgency lies in early diagnosis and personalized treatment. Immunotherapy's emergence propels cancer treatment into personalized precision medicine, utilizing molecular profiling, OMICS technology, biomarkers, and companion diagnostics for tailored, patient-specific approaches, building comprehensive databases for refined strategies (Deepa Jatti and Rakesh, 2021[22]).

### Biomarker identification

The mRNA expression in OSCC analysis took in studying 280 samples, including 19 normal tissues and 261 carcinoma tissues. By comparing gene expression between carcinoma and normal tissues, specific mRNAs linked to survival can be identified. Using Cox regression analysis and other bioinformatic methods, a unique 5-mRNA signature can be obtained that predicts carcinoma patient survival that shows promise as a potential biomarker for personalized cancer treatments (Guo et al., 2021[35]). In a study, bioinformatics analysis was employed to find biomarkers related to autophagy in OSCC. Autophagy plays a significant role in oral squamous carcinoma, and the prognostic value of autophagy-related genes was explored. By using R software, differential expression analysis was performed and established with a risk score model based on autophagy expressed genes. Through comprehensive bioinformatics analyses, potential autophagy-related biomarkers can be identified, which can later be validated in OSCC tissues and cell lines. The study successfully created a risk model that accurately predicts the prognosis of OSCC patients. Additionally, it has identified ATG12 and BID as two potential independent prognostic biomarkers, suggesting new therapeutic targets for OSCC (Huang et al., 2021[40]). Enhanceingsurvival by accessible screenings and transitioning to non-invasive brush biopsies of cytology in primary care, and using explainable AI accelerates diagnoses cost-effectively (Hirsch et al., 2023[39]). Risk strategies reveal glycoprotein biomarkers as aberrant expressions in oral cancer where AAT and APOA1 show promise as potential biomarkers, warranting clinical evaluation (Wong et al., 2021[111]). Nonetheless, Apo A-IV and LRG1 have been identified as robust oral cancer biomarkers, are valuable tools for screening and early diagnosis due to their efficacy (Chang et al., 2019[16]).

### Precision medicine in oral cancer

Precision medicine has emerged as a promising strategy in the field of oral cancer treatment, aiming to customize therapies according to the unique attributes of each patient, including their genetic makeup and the specific molecular profile of their cancer (Zhong et al., 2018[117]). This approach integrates various advanced technologies such as AI, genomics, proteomics, and bioinformatics to enhance the accuracy of diagnosis, prognosis, and treatment planning. The discovery of biomarkers that can forecast the course of the disease, how well a treatment will work, and the overall prognosis for patients with oral cancer is essential to precision medicine (Malcangi et al., 2023[63]). Researchers may recognize molecular fingerprints linked to certain subtypes of oral cancer by analyzing genomic and proteomic data, facilitating more accurate diagnostic and prognostic evaluations (Sá et al., 2021[88]; Su et al., 2021[95]). However, AI searches through enormous clinical and genetic data sets to find connections and patterns that human researchers might miss (Bi et al., 2019[11]; Quazi, 2022[82]). Thus, machine learning algorithms can predict treatment results, identify relevant biomarkers, and categorize patients into different risk groups (Glaab et al., 2021[33]). Additionally, AI-enabled imaging modalities such as CT, MRI, and PET permit precise delineation of tumor boundaries and early identification of oral cancer, thereby encouraging customized approaches to treatment (Paudyal et al., 2023[78]; Illimoottil and Ginat, 2023[43]). AI algorithms are being deployed more and more to develop personalized strategies to individuals diagnosed oral cancer, extending beyond diagnostic and imaging. AI assists doctors pick among the most effective therapy modalities, such as targeted drugs and immunotherapies, by assessing the distinctive molecular profile of each cancer patient by integrating genetic data with clinical features (Senthil Kumar et al., 2023[90]; Quazi, 2022[82]). Moreover, AI-powered prediction algorithms identify potential adverse consequences and the probability of a therapeutic response, permitting healthcare professionals to make choices on the best course of therapy and dosage. Precision medicine serves as essential for the successful treatment of oral cancer since it is capable of identifying abnormalities in genes and the molecular mechanisms for tumor development and metastasis (Krzyszczyk et al., 2018[57]). By deferring abnormal signaling processes or accounting of vulnerabilities in malignant cells, scientists are able to develop personalized therapies that steer clear of damage to healthy tissues by grasping the basic biology of oral cancer. Precision medicine offers significant promise to enhance the prognosis of patients with oral cancer via personalized diagnosis and treatment plans that address the distinctive characteristics of each patient as well as the biology of the tumor. Future advancements in AI, genomics, and other technologies appear to be contributing to more personalized and efficient treatment for oral cancer. 

## Limitations and Problematic results

### Ethical considerations

Stringent regulations must be implemented as the utilization of AI in healthcare poses an array of legal and ethical issues. Owing to the shortage of established regulations, employing AI in healthcare involves a number of hazards such as the likelihood of errors (Amann et al., 2020[7]), data security breaches, and patient damage (Zhang and Zhang, 2023[116]). One of the primary challenges is the fact that currently there are no clear regulations regulating the implementation/adoption of AI in healthcare settings (Esmaeilzadeh, 2020[28]). The absence of stringent rules might give rise to the unrestricted creation and execution of AI systems, that might give rise to errors of judgement, security vulnerabilities, and negative outcomes for patients, mistakes when interpreting AI-driven diagnosis and therapy recommendation. While AI algorithms are not perfect, they are capable of analyzing enormous amounts of data and find patterns that human beings are incapable of recognizing (Liu et al., 2022[61]; Cheng et al., 2021[18]). Inaccurate diagnosis or treatment plans harm patients, which emphasizes the significance it is to ensure that AI technology is accurate and trustworthy. Additionally, extremely personal information that is sensitive is incorporated in the integration of AI with healthcare data, requiring to be rigorously safeguarded from misuse or illicit access (Pool et al., 2024[80]). Patient information gathering and analysis is being managed by a growing number of AI systems, which increases the likelihood of data breaches that jeopardize confidentiality of patient information and undermine the public's trust in the health care sector. The concept of transparency, anonymity, and cybersecurity must come foremost in the advancement and implementation of AI technology in healthcare settings to address these ethical dilemmas. Healthcare providers and tech developers must be forthright about the positive and negative aspects of AI systems in order to guarantee that clients have complete knowledge about the use of their personally identifiable data and the risks involved (Fritsch et al., 2022[31]). Rigorous safety precautions must also be put into effect to safeguard patient data from exploitation or unlawful access. Encryption approaches, tight restrictions on access, and data methods for anonymization have to be adopted in order to mitigate the risk of breaches and unauthorized disclosure (Redrup Hill et al., 2023[85]). The establishment of complete code of conduct and regulatory structures that allow the accountable and ethical use of AI in healthcare practices will necessitate continuous dialogue and cooperation between a variety of stakeholders, notably patients, technological designers, lawmakers, and healthcare professionals.

A systematically executed study has observed that utilization of AI in regular clinical practice could be one of the potential drawbacks felt by most of the authors (Alabi et al., 2021[4]).

### Data privacy and security

In the era of digital healthcare and AI-powered diagnostics, protecting data security and privacy becomes crucial for boosting patient confidence in healthcare systems. Wearable technology and electronic health records (EHRs) are two examples of digital health tools that are increasingly in use. Given the huge quantities of private patient data produced, there are concerns regarding data security and privacy (Dinh-Le et al., 2019[24]; Kasoju et al., 2023[50]). One of the primary barriers for safeguarding data privacy is the immense and diversified volume of health-related information that gets generated and stored (El Mestari et al., 2024[25]). EHRs offer an extensive amount of personal health data, encompassing medical histories, treatment plans, and diagnosis outcomes which emphasizes on safeguarding against exposure or illegal access. Additionally, the possibility of cyberattacks and data breaches increases as a result of healthcare systems' interconnectivity (Javaid et al., 2023[47]). Cybercriminals may use loopholes in hospital IT systems to gain access to private patient data, jeopardizing patient privacy and placing patients in danger (Wasserman and Wasserman, 2022[107]). A comprehensive strategy encompassing organizational, technical, and regulatory measures requires implementation to tackle these issues. To protect patient information from unauthorized entry or manipulation, healthcare facilities need to set up security measures such as intrusion detection systems, access controls, and robust data encryption. Further, employee awareness-raising and education efforts have to occur first in order to foster a culture of data protection and privacy (Alshaikh, 2020[6]). This means imparting knowledge to employee on how to recognize and manage potential security threats like malware assaults and phishing scams. Lawmakers need to enact legislation and rules that establish rigorous standards for patient privacy and data security in healthcare environments (McGraw and Mandl, 2021[69]). It is essential for healthcare providers to strictly adhere to the established rules for encryption of data, breach alert, and accountability systems to guarantee that they comply with their responsibility to safeguard patient data. Throughout, coordination amongst various stakeholders including technology companies, patients, regulators, and healthcare providers is essential for safeguarding data security and privacy in the healthcare industry (Motti and Berkovsky, 2022[72]). Recently, the ChatGPT tool has been studied for understanding suggestions based on AI and explored the effectiveness in its efficacy in diagnosis and healthcare. Thus, AI has shown beneficial effects but still a comprehensive assessment and governance is warranted (Ahmed, 2024[3]). Healthcare organizations may minimize their risk of data breaches through establishing robust organizational and technical security measures in place and implementing legal requirements that safeguard the privacy and integrity of patients.

### Interpretability and trustworthiness

AI offers tremendous potential for transforming the healthcare sector; yet the most important fear is ensuring the dependability and interpretability of AI systems. While interpretability is the ability to understand and make sense of the decisions generated by artificial intelligence systems, trustworthiness is the consistency, accuracy, and objectivity of these judgements (Ennab and McHeick, 2022[26]; von Eschenbach, 2021[103]). The interpretability of AI is severely hampered by the inherent complexity of many machine learning models (Papadimitroulas et al., 2021[77]). In particular, deep learning algorithms often function as "black boxes," which makes it challenging to gain insight into the rationale behind their findings (Krishnan, 2020[56]). If prejudices are inadvertently preserved in the data that AI systems are educated on, healthcare results for particular demographic groups may vary (Varona and Suárez, 2022[101]). Additionally, the absence of transparency in AI decision-making procedures renders it more challenging to recognize and deal with instances of bias or discrimination (Nazer et al., 2023[75]; Tsamados et al., 2022[99]). Scientists and engineers have to place the greatest emphasis on developing coherent AI models that throw light on decision-making processes to address these issues. To improve comprehensibility and clarity, methods such as rule-based approaches, feature importance analysis, and model visualization are employed. Additionally, comprehensive testing, validation, and examination should be a part of the procedure that guarantees the reliability of AI systems and assesses their efficiency across a range of clinical settings and demographics (Markus et al., 2021[66]). The efficacy of the model must be assessed with representative datasets, and results must be continuously tracked to look for undesirable or unexpected outcomes and removed. Policies that encourage the ethical and responsible application of AI in healthcare must be developed by regulatory bodies and healthcare institutions. These policies should include processes for assessing and certifying AI algorithms as well as guaranteeing accountability for mistakes or unfavourable results (Markus et al., 2021[66]). Healthcare stakeholders can fully utilize AI's potential for improving patient care, support clinical decision-making, and advance medical research while upholding accountability, transparency, and equity by addressing these challenges while making interpretability and reliability top priority in AI development and implementation.

## Conclusion

In recent times, there has been advancement in utilizing AI to diagnose and predict the progression of oral cancer. Past research has demonstrated that machine learning yields precise outcomes in detecting oral cancer. It aids healthcare professionals in diagnostic procedures, reducing the risk of unintended mistakes. However, past research utilizing deep learning (neural network) has shown greater precision in the quick identification of oral cancer as compared to machine learning methods. Utilizing AI offers the potential to enhance detection exactness of oral cancers and OPMDs by integrating novel methods with conventional approaches. In addition, AI can aid in predicting the progression of precancerous and cancerous lesions based on retrospective data analysis. Future studies might explore the progress of data fusion algorithms that integrate diverse modalities like clinical, histological, radiological, and molecular evaluations to aid in the primary detection and prognosis assessment of the disease.

## Notes

Bhupendra G. Prajapati and Summya Rashid (Department of Pharmacology & Toxicology, College of Pharmacy, Prince Sattam Bin Abdulaziz University, P.O. Box 173, Al-Kharj 11942, Saudi Arabia; E-mail: s.abdulrashid@psau.edu.sa) contributed equally as corresponding author.

## Declaration

### Acknowledgment

This study is supported via funding from Prince Sattam Bin Abdulaziz University project number (PSAU/2024/R/1445). Gehan M. Elossaily would like to thank AlMaarefa University, Riyadh, Saudi Arabia for supporting this work. Dr. Prajapati extends his sincere appreciation to the Faculty of Pharmacy, Silpakorn University, Thailand, for their generous support that enabled the completion of this work.

### Ethics approval and consent to participate

N/A. 

### Consent for publication

All authors provided consent for publication.

### Conflict of interest 

The authors declare that they have no conflict of interest.

## Figures and Tables

**Table 1:**
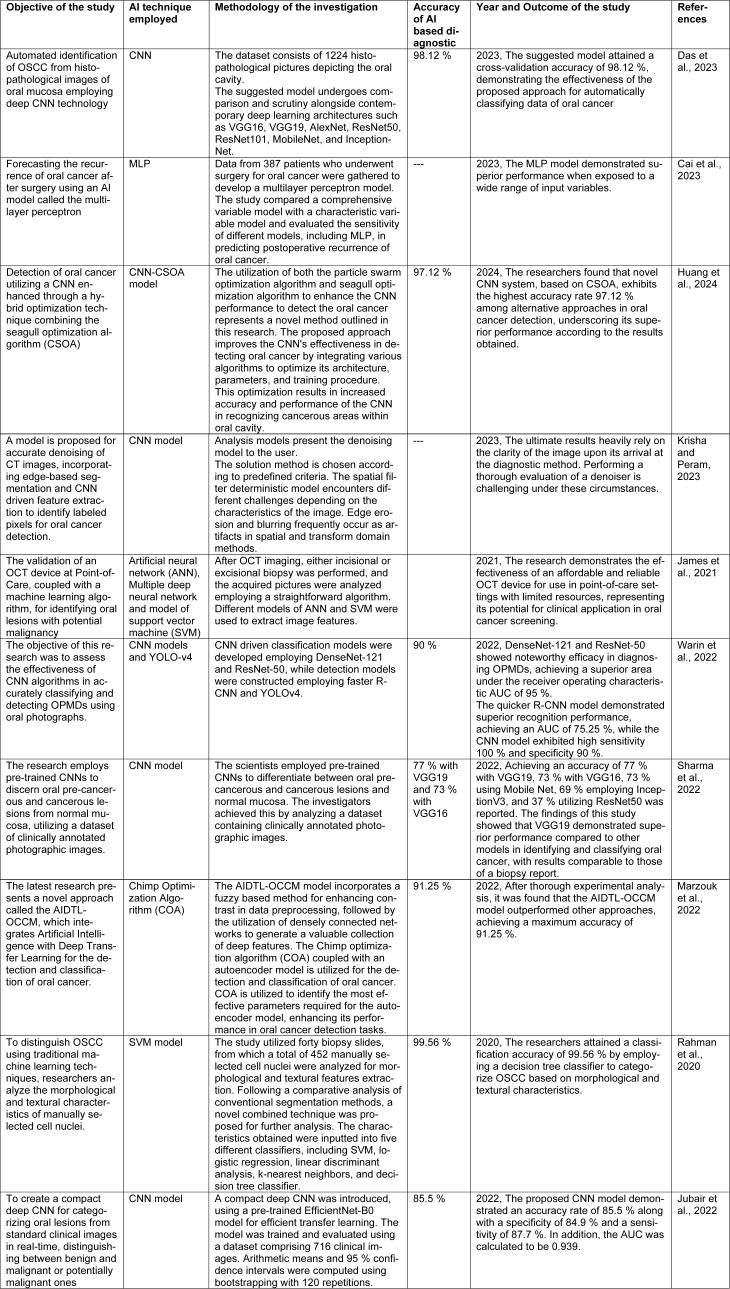
Different investigations utilizing AI in oral cancer have shown promise in early detection, diagnosis, and predicting treatment outcomes.

**Figure 1:**
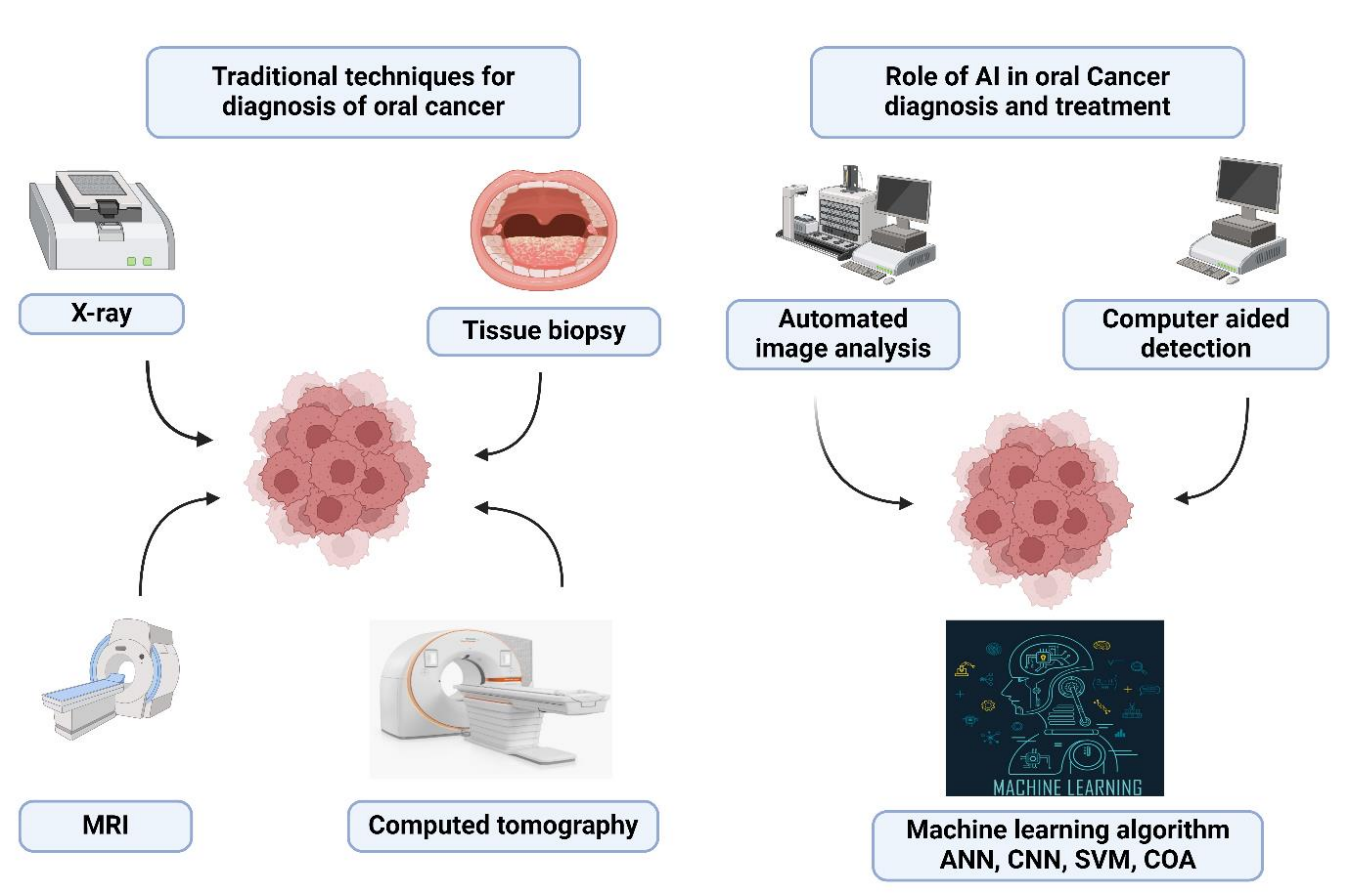
Graphical abstract
